# The Effect of the COVID-19 Vaccine on Daily Cases and Deaths Based on Global Vaccine Data

**DOI:** 10.3390/vaccines9111328

**Published:** 2021-11-15

**Authors:** Zhiwei Li, Xiangtong Liu, Mengyang Liu, Zhiyuan Wu, Yue Liu, Weiming Li, Mengmeng Liu, Xiaonan Wang, Bo Gao, Yanxia Luo, Xia Li, Lixin Tao, Wei Wang, Xiuhua Guo

**Affiliations:** 1 Department of Epidemiology and Health Statistics, School of Public Health, Capital Medical University, 10 Xi-Tou-Tiao, You-An-Men Street, Fengtai District, Beijing 100069, China; lizhiwei@ccmu.edu.cn (Z.L.); xiangtongl@ccmu.edu.cn (X.L.); ysq@mail.ccmu.edu.cn (M.L.); wuxiaozhi@ccmu.edu.cn (Z.W.); liuyue@ccmu.edu.cn (Y.L.); lwming@ccmu.edu.cn (W.L.); liumengmeng@ccmu.edu.cn (M.L.); xiaonanw@ccmu.edu.cn (X.W.); gaobo@ccmu.edu.cn (B.G.); lyx100@ccmu.edu.cn (Y.L.); taolixin@ccmu.edu.cn (L.T.); 2 Beijing Municipal Key Laboratory of Clinical Epidemiology, Capital Medical University, Beijing 100071, China; wei.wang@ecu.edu.au; 3 Department of Mathematics and Statistics, La Trobe University, Melbourne, VIC 3086, Australia; x.li2@latrobe.edu.au; 4 Center for Precision Health, Edith Cowan University, Perth, WA 6027, Australia

**Keywords:** COVID-19 cases, COVID-19 deaths, vaccination, prediction, worldwide

## Abstract

Background: Coronavirus disease 2019 (COVID-19), a global pandemic, has caused over 216 million cases and 4.50 million deaths as of 30 August 2021. Vaccines can be regarded as one of the most powerful weapons to eliminate the pandemic, but the impact of vaccines on daily COVID-19 cases and deaths by country is unclear. This study aimed to investigate the correlation between vaccines and daily newly confirmed cases and deaths of COVID-19 in each country worldwide. Methods: Daily data on firstly vaccinated people, fully vaccinated people, new cases and new deaths of COVID-19 were collected from 187 countries. First, we used a generalized additive model (GAM) to analyze the association between daily vaccinated people and daily new cases and deaths of COVID-19. Second, a random effects meta-analysis was conducted to calculate the global pooled results. Results: In total, 187 countries and regions were included in the study. During the study period, 1,011,918,763 doses of vaccine were administered, 540,623,907 people received at least one dose of vaccine, and 230,501,824 people received two doses. For the relationship between vaccination and daily increasing cases of COVID-19, the results showed that daily increasing cases of COVID-19 would be reduced by 24.43% [95% CI: 18.89, 29.59] and 7.50% [95% CI: 6.18, 8.80] with 10,000 fully vaccinated people per day and at least one dose of vaccine, respectively. Daily increasing deaths of COVID-19 would be reduced by 13.32% [95% CI: 3.81, 21.89] and 2.02% [95% CI: 0.18, 4.16] with 10,000 fully vaccinated people per day and at least one dose of vaccine, respectively. Conclusions: These findings showed that vaccination can effectively reduce the new cases and deaths of COVID-19, but vaccines are not distributed fairly worldwide. There is an urgent need to accelerate the speed of vaccination and promote its fair distribution across countries.

## 1. Introduction

Coronavirus disease 2019 (COVID-19) is a complex respiratory illness caused by SARS-CoV-2, a novel coronavirus first reported in Wuhan, China. COVID-19 is thus far the most severe pandemic of the 21st century, causing 176 million reported cases and 3.81 million deaths worldwide as of June 2021. SARS-CoV-2 has spread rapidly, overwhelming health systems and clinical management, and has been considered as an unprecedented public health challenge [1,2,3,4,5]. Therefore, strong preventive and control measures must be taken, such as social distancing, lockdown, wearing masks and maintaining a healthy lifestyle. Despite the actions taken, SARS-CoV-2 has had a profound impact on infected individuals as well as the entire population, negatively affecting the global economy and resources and leading to mental disorders, such as depression and anxiety [6,7]. Previous studies have shown that vaccine, as a powerful weapon to control an infectious disease, can notably allay or defeat diseases caused by pathogens [8,9]. There is an urgent need to develop vaccines to limit the spread of SARS-CoV-2 with the aim of achieving “herd immunity”.

Currently, several vaccines are in phase IV clinical trials, Pfizer/BioNTech, Moderna, AstraZeneca, Sinovac; and more than 200 vaccine candidates are also in their early clinical trials. Compared to previous vaccines, COVID-19 vaccine development has proceeded at an incredible pace and was quickly put into use owing to the coordinated effort between the scientific, medical community and government agencies [10]. The worldwide efforts to develop a safe and effective COVID-19 vaccine is extraordinary, and the vaccination research is now underway in many countries. However, due to the limited vaccine production capacity, the global supply of vaccines is in short condition. Moreover, with uneven levels of medical care and economic development across countries, there is a global imbalance distribution in vaccination [11].

Most studies on vaccine efficacy are clinical trial studies, which have strict conditions, and the calculated efficacy is only theoretical [12,13,14,15,16,17]. The most intuitive manifestation of vaccine effectiveness in the real world is the effect on the number of new cases and deaths of COVID-19, but no study has yet examined the association between COVID-19 vaccination and the numbers of new cases and deaths.

To address this knowledge gap, this study first provides comprehensive analyses of global vaccine delivery and then, after adjusting for multiple covariates, investigates the relationship between daily vaccination and the numbers of new cases and deaths per day to identify the effectiveness of the vaccination.

## 2. Material and Methods

### 2.1. Data Collection

#### 2.1.1. Vaccination Data

Vaccination data were derived from the Our World in Data website [18], a vaccination dataset that uses the most recent official numbers from government health ministries in each country worldwide. The beginning time point was selected based on the date of the first country starting its vaccinations, and the end time point was the date of completion of our analysis, i.e., vaccination data were collected from 20 December 2020 to 25 April 2021. If a country was not included, it means that no one in that country was vaccinated or no vaccination data were published during the time frame of this study. The dataset contains 59 variables. A detailed explanation of each variable can be found at https://github.com/owid/covid-19-data/blob/master/public/data/owid-covid-codebook.csv (accessed on 25 October 2021). The variables used in this analysis were the daily total number of people who received at least one vaccine dose, daily total number of people who received all doses prescribed by the vaccination protocol, daily new confirmed cases of COVID-19 and daily new deaths attributed to COVID-19. We used a snippet to illustrate the main variables:

Three people take part in a vaccination program, to be given a vaccine that requires 2 doses to be effective against the disease. Dina has received two doses; Tommy has received one dose; Ellie has not received any dose. In our data, the total number of people receiving at least one dose of vaccine will be equal to 2 (Dina, Tommy); The total number of people fully vaccinated will be equal to 1 (Dina). The total number of people not vaccinated will be equal to 0 (Ellie).

#### 2.1.2. Confounding Factors

Some studies have shown that meteorological factors, such as temperature, can affect vaccine efficacy and the motivation of the population to become vaccinated. For example, the stability of the vaccine will decrease at tropical ambient temperatures [19]. Outside the temperature range of 2–8 °C, the activity of the vaccine is greatly reduced [20]. In Alabama, USA, multiple health departments delayed vaccinations due to extreme weather, which led to a decrease in the population’s motivation to become vaccinated [21]. To control these confounding factors, meteorological data were collected from the Global Surface Summary of the Day (GSOD) https://www.ncei.noaa.gov/access/metadata/landing-page/bin/iso?id=gov.noaa.ncdc:C00516 (accessed on 25 October 2021), which includes global data obtained from the National Oceanic and Atmospheric Administration (NOAA). The daily mean temperature (°C, TEMP), dew point temperature (°C, DEWP), air pressure (kPa, STP), wind speed (m/s, WDSP) and precipitation (mm, PRCP) were calculated based on 26,531 fixed meteorological monitors globally.

### 2.2. Statistical Analysis

First, we described the distribution of vaccines in the first top 10 and the last top 10 countries around the world, including the time of vaccine initiation and vaccination rates in each country using aggregated vaccination data.

Then, we used a generalized additive model (GAM) to explore the relationship between daily vaccinated people and daily confirmed COVID-19 cases or deaths. GAM is an extension of the generalized linear model and can be used in time series analysis, dealing with the complex nonlinear relationship of various variables using a smooth function [22,23,24]. We calculated the percentage change (%, *PC*) in the number of daily cases or deaths with per 10,000 people receiving at least one dose of vaccine or being fully vaccinated for each country. We used the coefficients of the GAM model to calculate the *PC* value. The calculation formula is below:

PC=[exp(β0∗Δc)−1)]∗100

where *PC* indicates the percent change in the daily number of new cases or deaths, and β0 refers to the coefficient of daily people receiving at least one dose of vaccine or people becoming fully vaccinated from the GAM model. Δc is the unit increase in the number of daily people at least one dose vaccine or people fully vaccinated. In our current study, Δc was set to 10,000 to obtain an appropriate effect value.

Next, we used a two-stage analytic protocol to explore whether vaccination can affect the daily number of new COVID-19 cases or deaths. In the first stage, we determined the parameters of the GAM model. In the second stage, a random effect meta model was used to pool the *PC* values from each country. We then reported the pooled effect value and the corresponding 95% confidence interval (*CI*) as the *PC* value in the daily number of cases or deaths per 10,000 people vaccinated or fully vaccinated. More specifically, we first selected the link function. Considering that all dependent variables are counting data, we chose the Poisson distribution as the link function of GAM. Second, we defined the degree of freedom (*df*) of the time trend. In the base model, only the time trend controlled with a restricted cubic spline was included, and we calculated the AIC (Akaike information criterion) value based on different *df* of the time trend. The *df* with the minimum AIC value was defined as the best *df* for the time trend. Third, we put other covariates into the model. In brief, holiday and day of the week were put into the model as factors to eliminate the holiday effect. Meteorological variables, such as TEMP, DEWP, STP, WDSP and PRCP, were included in the model using a restricted cubic spline with the best *df* to control for confounding factors. Fourth, independent variables (daily people receiving at least one dose of vaccine or fully vaccinated) were included in the model. The GAM model fitted in this study is shown as follows:

$$logE\left(Y_{t}\right)=\alpha +\beta Z_{t}+s\left(time,\;7\right)+s\left(TEMP,\;4\right)+s\left(DEWP,\;4\right)+s\left(STP,\;4\right)+s\left(WDSP,\;4\right)\;+s\left(PRCP,\;4\right)+facotr\left(DOW_{t}\right)+factor\left(Holiday_{t}\right)$$

where $E\left(Y_{t}\right)$ is the expected number of daily cases or deaths at Day *t*; α is the intercept; $Z_{t}$ denotes the number of people receiving at least one dose of vaccine or fully vaccinated at Day *t*; *s*() refers to the restricted cubic spline; and *TEMP*, *DEWP*, *STP*, *WDSP* and *PRCP* denote daily temperature, dew of point, air pressure and precipitation, respectively. *Dow* is an indicator of the day of the week, and *Holiday* is an indicator of the holiday effect. All the numbers in the model represent the best *df* in the restricted cubic spline function.

Next, we used two different lag structures to explore the lag effect of vaccination on the daily number of new cases or deaths. A single lag is expressed as lag1, lag2, …, lag30, where lag1 indicates that the daily number of cases or deaths in the current day is affected by the number of vaccine people in the previous day. Cumulative lag is shown as lag01, lag02, …, lag30, where lag01 refers to the daily number of deaths in the current day being affected by the average number of vaccinated people in the previous day and the current day. Considering that the vaccine becomes effective 3–4 weeks after injection [16], we chose a maximum lag period of 30 days.

Finally, in addition to building the GAM model using data from 187 countries worldwide, we also fit the GAM model using data from the United States alone. Since the daily number of new cases and deaths in the United States was maintained at a certain level during the study period (the average numbers of daily new cases and deaths were 112,679 and 1977, respectively), and the daily number of vaccinated people was also large (the average numbers of fully vaccinated people and people with at least one dose of vaccine were 917,360 and 1,104,708, respectively), the fitted model was highly reliable, and its results can provide a reference for vaccine distribution and administration in other countries.

In this study, R-4.0.3 software was used for basic statistical description and data cleaning. The “MGCV” and “Meta” packages in R software were used to fit the GAM model. A two-sided test was used in all statistical analyses, and the test level was *α* = 0.05. *p* < 0.05 was considered statistically significant.

The study protocol was approved by the Institutional Review Board at the School of Public Health, Capital Medical University. Written informed consent was not required because we used aggregated data and no individualized data.

### 2.3. Data Availability

The data that support the findings of this study are available at https://ourworldindata.org/covid-vaccinations and https://www.ncei.noaa.gov/access/metadata/landing-page/bin/iso?id=gov.noaa.ncdc:C00516 (accessed on 25 October 2021).

## 3. Results

### 3.1. Global Vaccination Overview

In total, 187 countries and regions were included in the study, and the study time ranged from 20 December 2020 to 25 April 2021. During the study period, 1,011,918,763 doses of vaccine were administered, 540,623,907 people received at least one dose of vaccine, and 230,501,824 people received two doses (see Supplementary: original country data.xlsx). In this study, the first two countries to begin vaccination were the United States and Israel on 20 December 2020, and the last five countries to begin vaccination were Djibouti, Libya, Lesotho, Niger and Somalia on 17 April 2021 (see Table 1 and Table S1).

In this study, the top 10 countries with the highest total number of vaccines administered cumulatively accounted for 76.06% of the total number of vaccines administered globally. The country with the highest number of vaccines injected was the United States (225,640,460), accounting for 22.30% of the total number of vaccines worldwide, followed by China (220,309,000) and India (138,379,832), which accounted for 21.78% and 13.67% of the total number of vaccines administered worldwide, respectively, while the fourth place, the United Kingdom (45,580,400), accounted for only 4.50%.

The 10 countries with the lowest number of vaccines were mostly developing countries in Africa and Oceania, with a cumulative percentage of 0.94 per 100,000, indicating a highly uneven distribution of vaccines globally (see Table 1).

In the current study, some controversial regions (e.g., Gibraltar and Falkland Islands) were counted as individual vaccine injections, which resulted in some areas with smaller populations having a higher proportion of fully vaccinated population; thus, we screened countries or regions with a population size greater than 100,000 for statistical description (see Table S2).

In terms of the proportion of the fully vaccinated population relative to the total population, Israel ranked in first place, with 57.86% of the total population fully vaccinated. The second-ranked country was the United Arab Emirates with 38.79%, followed by Chile with 32.24%. Although the vaccination rate in the United States ranked the fifth among the top 10 countries, the total population in the US is 38.24 times larger than that in Israel, which ranked the first. It is a remarkable achievement for the United States to have fully vaccinated 28.12% of the total population. After screening other countries with the similar population size as the United States (100 million or more people, excluding China because there are no data on the fully vaccinated population), we can see that the ratio of the fully vaccinated people to the total population in Brazil, which ranked the second, is only 5.20%, while the ratio in the United States is 5.4 times higher than that in Brazil (see Table S3).

All 10 countries with the lowest proportion of fully vaccinated people to the total national population had a proportion of less than 1%, and most of them were in Asia and Africa.

#### 3.1.1. The Association between Vaccination and Daily Cases and Deaths for COVID-19 Globally

**Figure 1:**
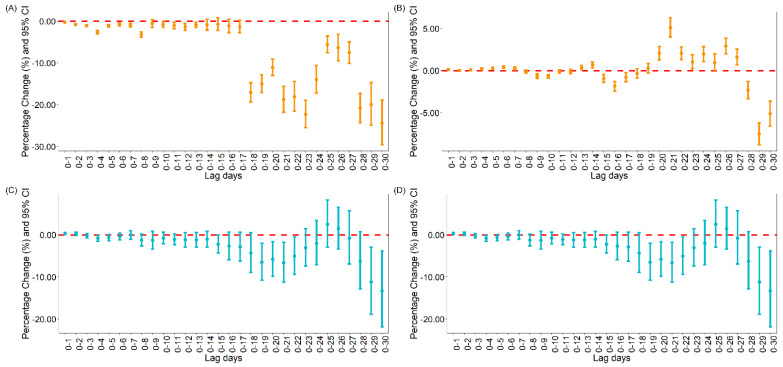
Percentage change of daily cases and deaths with moving lag globally. (**A**) Percentage change of daily cases with daily 10,000 people fully vaccinated increasing; (**B**) Percentage change of daily cases with daily 10,000 new people vaccinated increasing; (**C**) Percentage change of daily deaths with daily 10,000 people fully vaccinated increasing; (**D**) Percentage change of daily deaths with daily 10,000 people vaccinated increasing.

The GAM results showed a significant lag effect between vaccination and COVID-19 daily new cases or deaths both under the single lag model and moving lag model (see Figure 1 and Figure S1).

For the relationship between vaccination and new COVID-19 cases, the results showed that the daily new cases of COVID-19 would be reduced by 24.43% [95% CI: 18.89, 29.59] and 7.50% [95% CI: 6.18, 8.80] with 10,000 people per day becoming fully vaccinated and 10,000 people per day with at least one dose of vaccine at lag0–30 and lag0–29 under the moving lag model, respectively.

For the relationship between vaccination and new COVID-19 deaths, the results showed that the daily new deaths of COVID-19 would be reduced by 13.32% [95% CI: 3.81, 21.89] and 2.02% [95% CI: 0.18, 4.16] with 10,000 people per day becoming fully vaccinated and 10,000 people per day with at least one dose of vaccine both at lag0–30 under moving lag model, respectively. Similar results were also found under the single lag model (see Figure S1).

#### 3.1.2. The Association between Vaccination and Daily Cases and Deaths for COVID-19 in the United States

**Figure 2:**
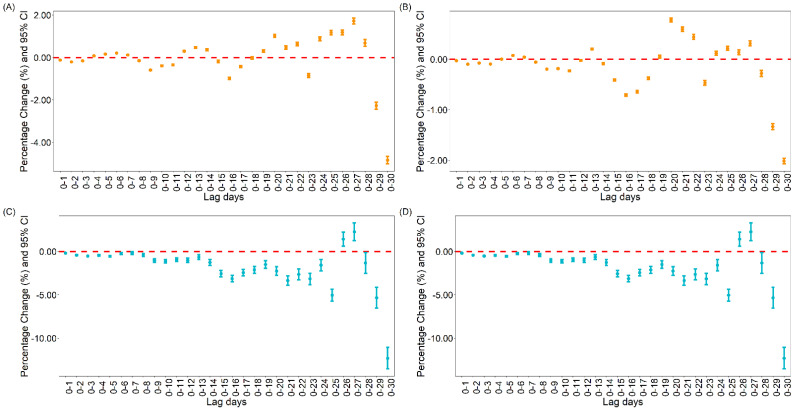
Percentage change in daily cases and deaths with moving lag in the US. (**A**) Percentage change of daily cases with daily 10,000 people fully vaccinated increasing; (**B**) Percentage change of daily cases with daily 10,000 people vaccinated increasing; (**C**) Percentage change of daily deaths with daily 10,000 people fully vaccinated increasing; (**D**) Percentage change of daily deaths with daily 10,000 people vaccinated increasing.

For the relationship between vaccination and new COVID-19 cases in the United States, the results showed that 10,000 fully vaccinated people per day and 10,000 people per day with at least one dose of vaccine would reduce the new COVID-19 cases by 4.84% [95% CI: 4.66, 5.02] and 2.02% [95% CI: 1.96, 2.07] both at lag0–30 under the moving lag GAM model, respectively (see Figure 2).

For the relationship between vaccination and new COVID-19 deaths in the United States, the results showed that 10,000 fully vaccinated people per day and 10,000 people per day with at least one dose of vaccine would reduce the new COVID-19 deaths by 12.31% [95% CI: 11.06, 13.53] and 3.36% [95% CI: 2.97, 3.76], respectively, at lag0–30 under the moving lag GAM model. Similar results were found under the single lag model (see Figure S3).

#### 3.1.3. Daily Cases and Deaths Predicted for COVID-19 with 10,000 Fully Vaccinated People

**Figure 3:**
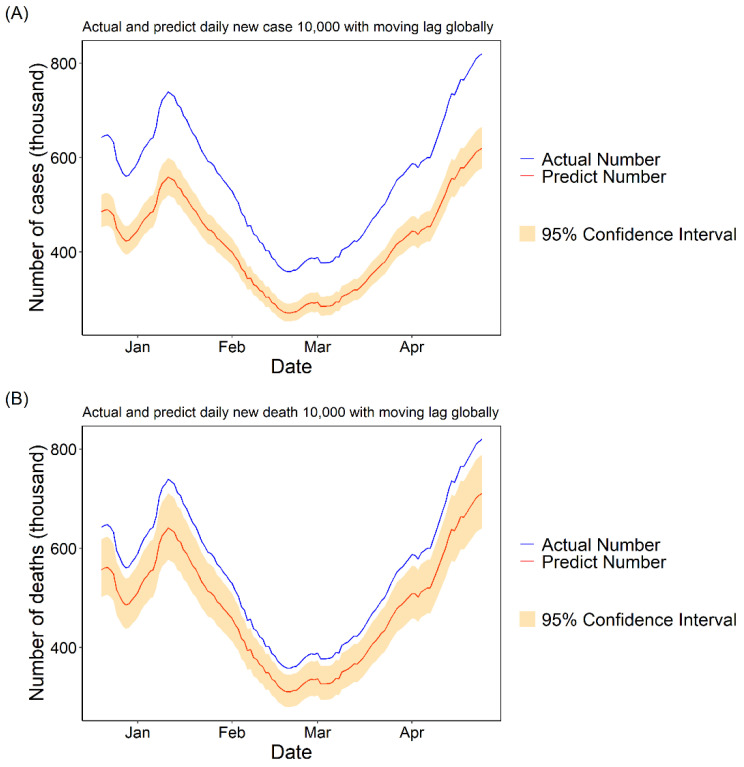
The actual and predicted daily cases and deaths numbers with a moving lag globally. The predicted number was calculated by multiplying the actual number of daily cases by the PC value of the best lag. (**A**) The PC value and related 95% CI are −24.43 [−29.59, −18.89] with the best moving lag of 30. (**B**) The PC value and related 95% CI are −13.32 [−21.89, −3.81] with the best moving lag of 30.

**Figure 4:**
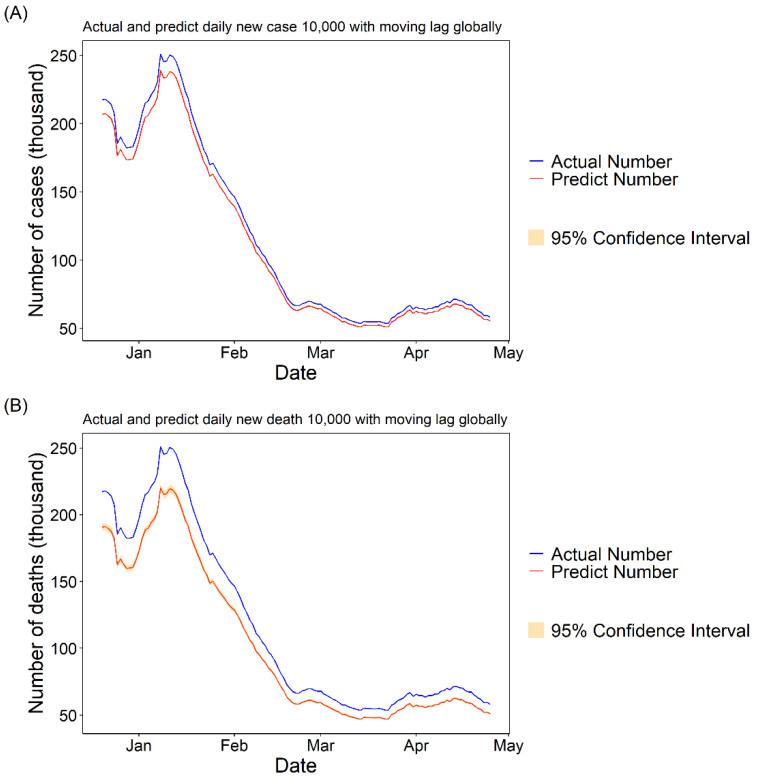
The actual and predicted daily cases and deaths numbers in the US. The predicted number was calculated by multiplying the actual number of daily cases by the PC value of the best lag. (**A**) The PC value and related 95% CI are −4.84 [−5.02, −4.66] with the best moving lag of 30. (**B**) The PC value and related 95% CI are −12.31 [−13.53, −11.06] with the best moving lag of 30.

To visualize the results of the GAM model intuitively, we used the *PC* values calculated from the moving lag GAM model multiplied by the true daily cases and deaths of COVID-19 to show the theoretical daily cases and deaths of COVID-19 if 10,000 people had received two doses of vaccine per day during the study period (we considered those who received two doses of vaccine to be fully immunized). The results showed that there would be a significant decrease in new COVID-19 cases and deaths per day both in the United States and globally (see Figure 3 and Figure 4).

## 4. Discussion

Our study began with the describing vaccination in 187 countries and regions worldwide, including people who received at least one dose of vaccine and two doses of vaccine (fully vaccinated people). Then, we used the GAM model to explore the relationship between daily vaccinated people and COVID-19 cases and deaths both globally and in the USA. Finally, we used the PC values calculated from the moving lag GAM model to predict the theoretical daily cases and deaths of COVID-19 if 10,000 people had received two doses of vaccine per day during the study period.

We found that vaccines were very unevenly distributed across countries globally. The top 10 countries with the highest total vaccine injections cumulatively accounted for 76.06% of the total global vaccine injections, while the bottom 10 countries accounted for less than 1% of total vaccine injections. As a result, some countries have distributed the second dose of vaccine to those who have never been vaccinated in order to deliver the first vaccination to as many people as possible. The practice of devoting a limited amount of first-dose vaccine to a larger number of people reduces the infection rate of COVID-19 to a certain extent, but in the long run, delivering only a single dose of vaccine may promote virus mutations [25]. The rate at which a virus mutates is closely related to the human body’s immune response. The less stress the immune response gives, the faster the virus adapts. If the immunity of some individuals is insufficient to inhibit viral replication, it may induce a variant with “immune escape” potential [25].

Harvard predicted that the human pandemic of COVID-19 could not end in a short term [26]. Vaccination has played a central role in the COVID-19 pandemic, especially in stopping new infections and saving millions of lives [14,27,28,29,30]. In contrast with single-dose vaccines, all approved coronavirus vaccine shots need to be injected as two doses, spaced apart. While the first dose helps build a sufficient immune response in the body, the second dose helps strengthen it and generates memory B cells that ‘remember’ the response. Thus, only after a person receives two full doses of the vaccine in a given timeline, is he or she considered to be fully vaccinated and protected. For economically developed countries, the vaccination rate is relatively high, especially for countries such as the United States, where the proportion of the population receiving two doses of vaccine is 5.4 times higher than that of the second-place country, Israel. Pandemic prevention and control are not a national or a regional effort; once a supervirus emerges during the spread of a pandemic, all humanity will be affected. To effectively contain the spread of COVID-19 globally, large economically developed countries need to help small countries so that we can end the epidemic as soon as possible.

Our study confirmed that the COVID-19 vaccine continues to work well in the real world, except in rigorous clinical trials, filling a gap in relevant research [17,27,30,31]. We found that with every increase of 10,000 fully vaccinated people, new cases would be reduced by 24.43% [95% CI: 18.89, 29.59] and new deaths would be reduced by 13.32% [95% CI: 3.81, 21.89] per day. The prediction curves also show that if 10,000 people per day received two doses of the vaccine, there would be a significant reduction in the daily number of new COVID-19 cases and deaths per day. Thanks to the high efficiency of the various vaccines, vaccination was able to significantly reduce the number of daily COVID-19 cases and deaths. The minimum effectiveness of the COVID-19 vaccine has been defined as 50% after discussions among WHO experts [32], and the currently marketed vaccines have higher than expected effectiveness of 95% for the Pfizer-BioNTech vaccine [16], 62.1% for Oxford-AstraZeneca [33], 94.1% for Moderna [34] and 91.6% for Gamaleya [35], among others.

Although we did not have access to the specific type of vaccines given to each individual in the current study, we still believe that our results have a high level of reliability. Inactivated virus vaccines, adenovirus-based vaccines and mRNA-based vaccines are the main available vaccines, but their immune response mechanisms are different [36]. Currently, vaccination is administered globally by giving the same type of vaccine, but some scholars are also exploring novel vaccination strategies, such as combining different types of COVID-19 vaccines, also known as heterologous vaccination, by which the vaccinated population may achieve better immunogenicity [37]. The mechanism of action has been elucidated through relevant studies, showing that heterologous vaccination enhances cytotoxic T-cell and Th1-activated immune responses [38]. Therefore, a clinical trial called ComCOV comparing combinations of COVID-19 vaccine schedules is underway to assess whether there are differences in the effectiveness of heterologous and homologous vaccination with COVID-19 vaccines [39]. These results will shed light on possible approaches regarding COVID-19 vaccination.

Our study has some strengths. First, we included 187 countries and regions worldwide, and the large scope of the study allowed us to provide a more comprehensive picture of the relationship between daily vaccinations and COVID-19 cases and deaths. Then, we used a two-stage analytic protocol method that gave a more accurate result. The GAM model used in stage one could deal with the complex nonlinear relationship of various variables in a time series analysis. The random effect meta model used in stage two aggregated the effect values for each country according to different weights. There are also limitations that need to be addressed. First, the study period was 128 days from 20 December 2020, to 25 April 2021, and longer continuing vaccination monitoring would enable collection of a more stable time series. Third, due to the lack of age- or sex-stratified datasets, we could not observe more significant results in more detailed stratifications at the population level.

## 5. Conclusions

In summary, for the first time, the results of our analysis show that vaccination can effectively reduce the new cases and deaths of COVID-19 in the real world, but vaccines are not evenly distributed across countries. Our study provides a theoretical basis for global COVID-19 prevention and control efforts; that is, we must accelerate the speed of vaccination and promote its fair distribution worldwide to eliminate the COVID-19 pandemic as quickly as possible.

## Figures and Tables

**Table 1:** The first and last top 10 countries of percentage of vaccine doses worldwide.

	Country Abbr.	Country Name	Continent	Total Vaccinations	Proportion (%)
First top 10	USA	United States	North America	225,640,460	22.30
	CHN	China	Asia	220,309,000	21.78
	IND	India	Asia	138,379,832	13.67
	GBR	United Kingdom	Europe	45,580,400	4.50
	BRA	Brazil	South America	37,730,651	3.72
	DEU	Germany	Europe	24,821,527	2.45
	TUR	Turkey	Asia	21,068,403	2.08
	FRA	France	Europe	19,225,460	1.90
	IDN	Indonesia	Asia	18,322,578	1.81
	RUS	Russia	Europe	18,080,498	1.79
Last top 10	NRU	Nauru	Oceania	168	1.66 × 10^−5^
	CMR	Cameroon	Africa	400	4.00 × 10^−5^
	TON	Tonga	Oceania	500	4.94 × 10^−5^
	ARM	Armenia	Asia	565	5.58 × 10^−5^
	LBY	Libya	Africa	750	7.41 × 10^−5^
	SSD	South Sudan	Africa	947	9.36 × 10^−5^
	PNG	Papua New Guinea	Oceania	1081	1.07 × 10^−4^
	NER	Niger	Africa	1366	1.35 × 10^−4^
	MSR	Montserrat	North America	1751	1.73 × 10^−4^
	SLB	Solomon Islands	Oceania	2000	1.98 × 10^−4^

Abbr.: ISO 3166-1 alpha-3-three-letter country codes.

## Data Availability

Publicly available datasets were analyzed in this study. This data can be found here: https://ourworldindata.org/covid-vaccinations (accessed on 25 October 2021).

## Supplementary Materials

The following are available online at https://www.mdpi.com/article/10.3390/vaccines9111328/s1, Table S1: The first and last top 10 countries to start vaccinating, Table S2: Top 10 countries in proportion of fully vaccinated with population more than 100,000, Table S3: Proportion of fully vaccinated with population more than 100,000,000, Table S4: Top 20 countries and regions in proportion of fully vaccinated, Figure S1: Percentage change of daily cases and deaths with single lag globally, Figure S2: The actual and predict* daily cases and deaths number with single lag globally, Figure S3: Percentage change of daily cases and deaths with single lag in US, Figure S4: The actual and predict* daily cases and deaths number in US.
