# Supplementary material for: The Effect of the COVID-19 Vaccine on Daily Cases and Deaths Based on Global Vaccine Data
Source: Vaccines (Basel). 2021 Nov 15;9(11):1328. doi: 10.3390/vaccines9111328 (PMC8622191; doi:10.3390/vaccines9111328)
Supplement: Supplementary file 1 [file vaccines-09-01328-s001.zip › Supplementary.pdf]

Table S1: The first and last top 10 countries to start vaccinating

	Country abbr	Country name	Continent	Start date	People vaccinated
First top 10	ISR	Israel	Asia	2020-12-20	7435
	USA	United States	North America	2020-12-20	556208
	RUS	Russia	Europe	2020-12-22	52000
	BHR	Bahrain	Asia	2020-12-23	38965
	CHL	Chile	South America	2020-12-24	420
	MEX	Mexico	North America	2020-12-24	2924
	AUT	Austria	Europe	2020-12-27	732
	CZE	Czechia	Europe	2020-12-27	1251
	DEU	Germany	Europe	2020-12-27	24494
	DNK	Denmark	Europe	2020-12-27	6375
Last top 10	DJI	Djibouti	Africa	2021-04-17	10246
	LBY	Libya	Africa	2021-04-17	750
	LSO	Lesotho	Africa	2021-04-17	16000
	NER	Niger	Africa	2021-04-17	1366
	SOM	Somalia	Africa	2021-04-17	117567
	TON	Tonga	Oceania	2021-04-16	500
	FJI	Fiji	Oceania	2021-04-15	27705
	SSD	South Sudan	Africa	2021-04-15	947
	ZMB	Zambia	Africa	2021-04-15	106
	COG	Congo	Africa	2021-04-14	14297

Country abbr: ISO 3166-1 alpha-3 – three-letter country codes

Table S2: Top 10 countries in proportion of fully vaccinated with population more than 100000

Country abbr	Country name	Continent	People fully vaccinated	Population	Proportion(%)
ISR	Israel	Asia	5008438	8655541	57.86
ARE	United Arab Emirates	Asia	3836521	9890400	38.79
CHL	Chile	South	6162713	19116209	32.24
BHR	Bahrain	Asia	516480	1701583	30.35
USA	United States	North	93078040	331002647	28.12
JEY	Jersey	Europe	27772	101073	27.48
MLT	Malta	Europe	99574	441539	22.55
SRB	Serbia	Europe	1303093	6804596	19.15
ABW	Aruba	North	19928	106766	18.67
GBR	United Kingdom	Europe	12071810	67886004	17.78

Country abbr: ISO 3166-1 alpha-3 – three-letter country codes

Table S3: Proportion of fully vaccinated with population more than 100000000

Country abbr	Country name	Continent	People fully vaccinated	Population	Proportion(%)
USA	United States	North	93078040	331002647	28.12
BRA	Brazil	South	11042940	212559409	5.20
RUS	Russia	Europe	6767873	145934460	4.64
MEX	Mexico	North	5646105	128932753	4.38
IDN	Indonesia	Asia	6699327	273523621	2.45
IND	India	Asia	21443345	1380004385	1.55
BGD	Bangladesh	Asia	2155296	164689383	1.31
JPN	Japan	Asia	849051	126476458	0.67
PHL	Philippines	Asia	214792	109581085	0.20

Country abbr: ISO 3166-1 alpha-3 – three-letter country code

Table S4: Top 20 countries and regions in proportion of fully vaccinated

Country abbr	Country name	continent	People fully vaccinated	Population	Proportion(%)
GIB	Gibraltar	Europe	31999	33691	94.97789
ISR	Israel	Asia	5008438	8655541	57.86395
SYC	Seychelles	Africa	54668	98340	55.59081
FLK	Falkland Islands	South America	1775	3483	50.96181
CYM	Cayman Islands	North America	28988	65720	44.10834
ARE	United Arab Emirates	Asia	3836521	9890400	38.79035
BMU	Bermuda	North America	22243	62273	35.71853
CHL	Chile	South America	6162713	19116209	32.23815
BHR	Bahrain	Asia	516480	1701583	30.35291
USA	United States	North America	93078040	331002647	28.12003
MCO	Monaco	Europe	10859	39244	27.67047
JEY	Jersey	Europe	27772	101073	27.47717
TCA	Turks and Caicos Islands	North America	10000	38718	25.82778
SMR	San Marino	Europe	8657	33938	25.50828
MLT	Malta	Europe	99574	441539	22.55158
GGY	Guernsey	Europe	13618	67052	20.30961
SRB	Serbia	Europe	1303093	6804596	19.15019
ABW	Aruba	North America	19928	106766	18.66512
IMN	Isle of Man	Europe	15657	85032	18.41307
GBR	United Kingdom	Europe	12071810	67886004	17.78247

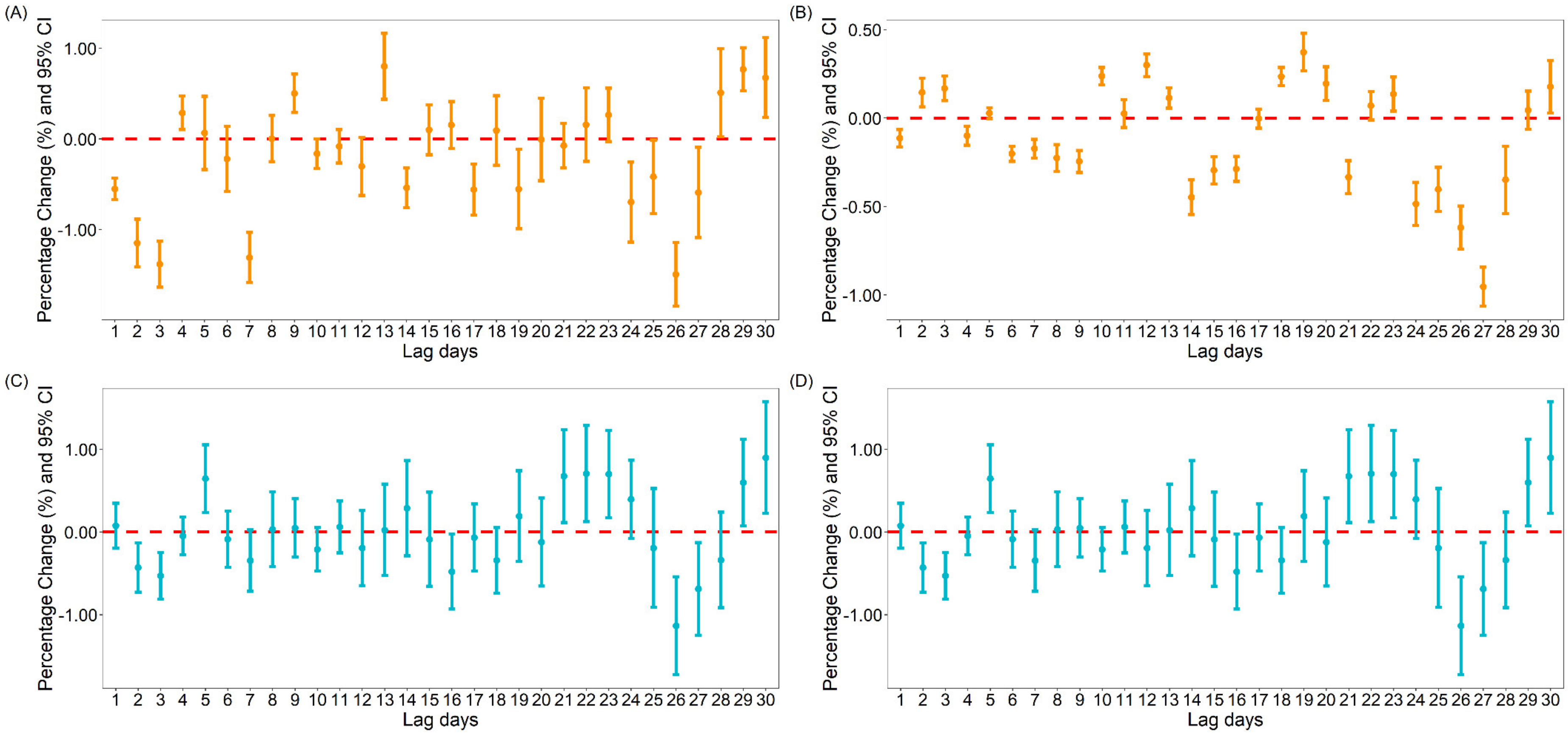

Figure S1: Percentage change of daily cases and deaths with single lag globally.

(**A**): Percentage change of daily cases with daily 10 thousand people fully vaccinated increasing;

(**B**): Percentage change of daily cases with daily10 thousand people vaccinated increasing;

(**C**): Percentage change of daily deaths with daily 10 thousand people fully vaccinated increasing;

(**D**): Percentage change of daily deaths with daily10 thousand people vaccinated increasing;

(A)

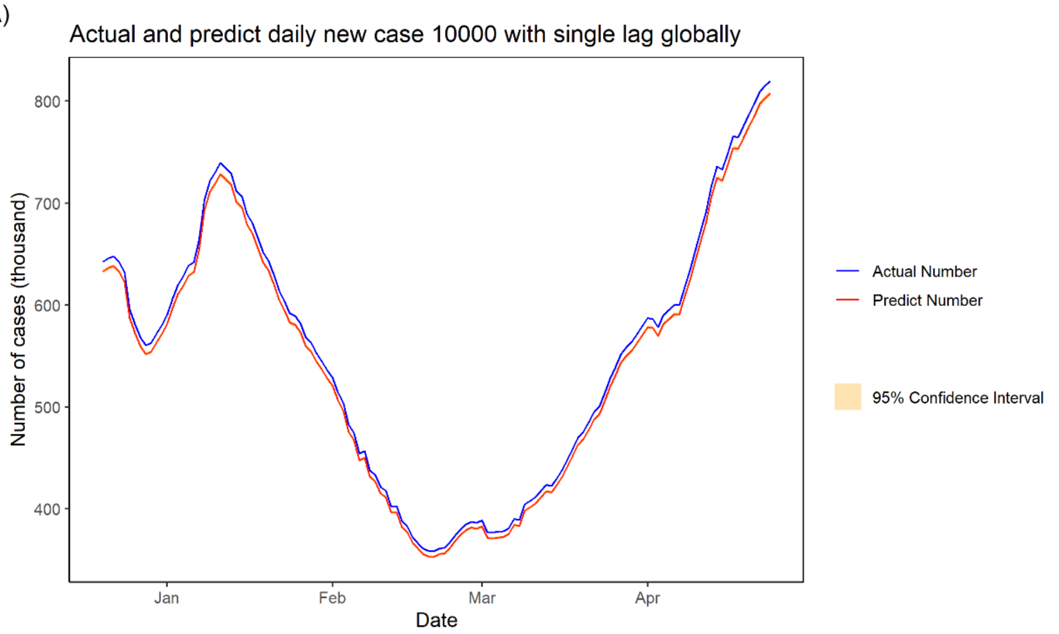

(B)

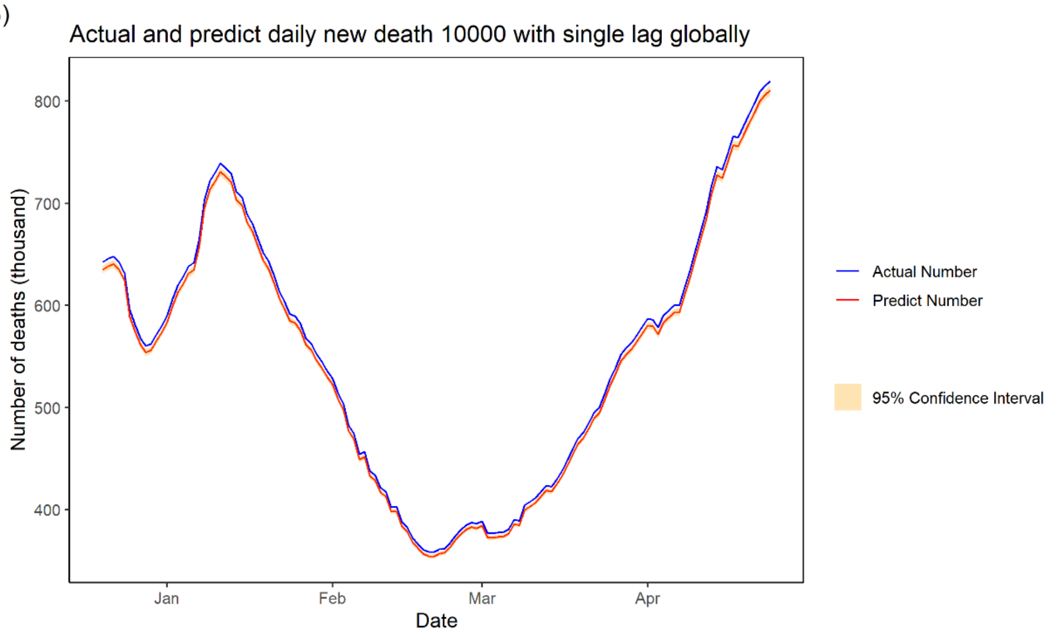

Figure S2: The actual and predict daily cases and deaths number with single lag globally.

The predict number was calculated by multiplying the actual number of daily cases by the PC value of the best lag.

(**A**): The PC value and related 95%CI is -1.50 [-1.85, -1.43] with best single lag of 26.

(**B**): The PC value and related 95%CI is -1.14 [-1.73, -0.54] with best single lag of 26.

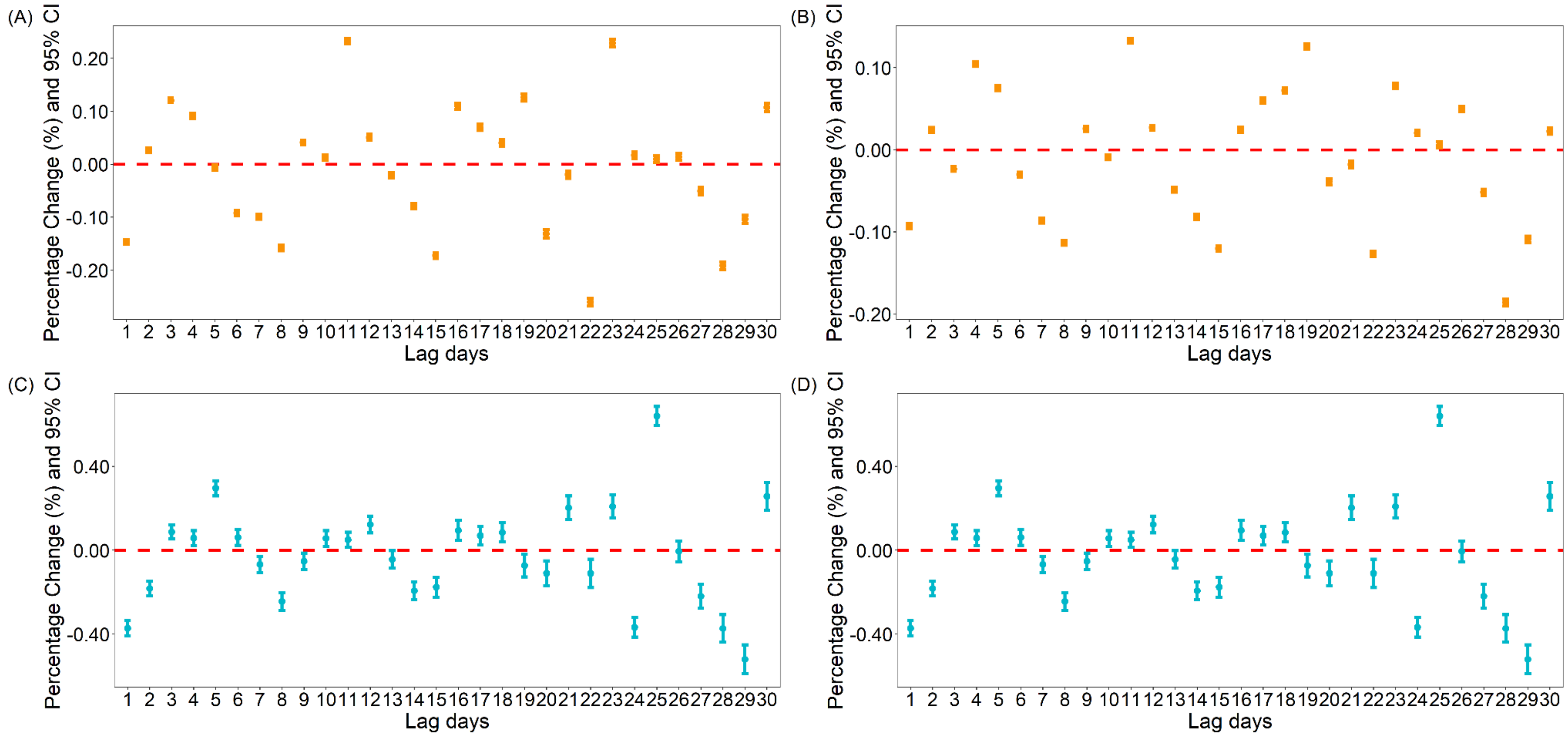

Figure S3: Percentage change of daily cases and deaths with single lag in US.

(**A**): Percentage change of daily cases with daily 10 thousand people fully vaccinated increasing;

(**B**): Percentage change of daily cases with daily10 thousand people vaccinated increasing;

(**C**): Percentage change of daily deaths with daily 10 thousand people fully vaccinated increasing;

(**D**): Percentage change of daily deaths with daily10 thousand people vaccinated increasing;

(A)

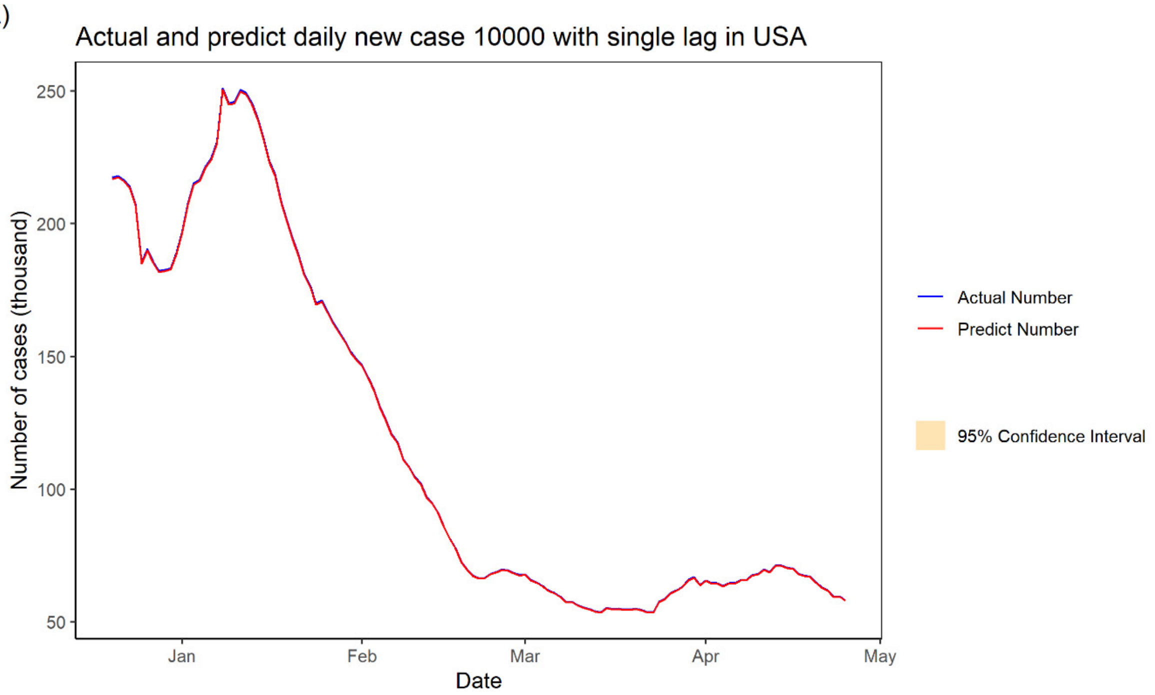

(B)

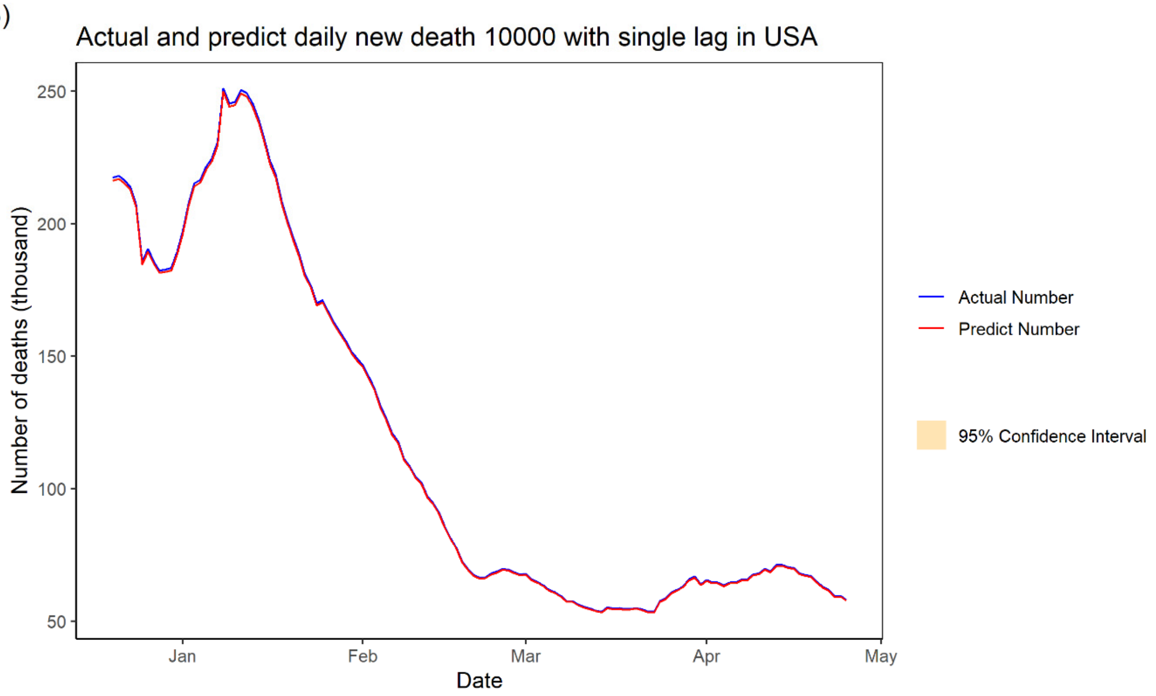

Figure S4: The actual and predict daily cases and deaths number in US.

The predict number was calculated by multiplying the actual number of daily cases by the PC value of the best lag.

(**A**): The PC value and related 95%CI is -0.26 [-0.27, -0.25] with best single lag of 22.

(**B**): The PC value and related 95%CI is -0.52 [-0.59, -0.45] with best single lag of 29.
